# Combination Therapy With Erlotinib and Ramucirumab in a Patient With Epidermal Growth Factor Receptor Mutation Positive Lung Adenocarcinoma and Interstitial Pneumonia: A Case Report

**DOI:** 10.7759/cureus.49528

**Published:** 2023-11-27

**Authors:** Shunya Tanaka, Taisuke Tsuji, Kazuki Jinno, Shoki Matsumoto

**Affiliations:** 1 Department of Respiratory Medicine, Japanese Red Cross Society Kyoto Daiichi Hospital, Kyoto, JPN

**Keywords:** anti-ars antibody, nonspecific interstitial pneumonitis, anti-vegf inhibitor, egfr-tki, non-small cell lung adenocarcinoma

## Abstract

Interstitial pneumonia often acts as a barrier to lung cancer treatment. We report the case of a 79-year-old man who was diagnosed with epidermal growth factor receptor (EGFR) mutation positive lung adenocarcinoma (T2aN0M0, stage ⅠB, EGFR exon 19 deletion), and was positive for anti-aminoacyl-tRNA synthetase antibodies with interstitial pneumonia. Metastasis in the right seventh rib was detected three months after surgical resection and radiation therapy was initiated. As recurrence was observed at both ends of the radiation field five months later, combination chemotherapy with erlotinib and ramucirumab was initiated. Approximately one year has passed since the start initiation of treatment, and acute exacerbation of interstitial pneumonia has not been observed during the follow-up period observation. The tumor has remained stable, indicating stable disease.

## Introduction

Non-small cell lung cancer (NSCLC) accounts for nearly 85% of cases of primary lung cancer. Most patients with NSCLC present with advanced stage of disease at the time of diagnosis [1]. Epidermal growth factor receptor (EGFR) mutation driven NSCLC occurs in approximately 10-20% of white patients and 40-60% of Asian patients [2]. Approximately 90% of EGFR mutations may be attributed to deletion within exon 19 (ex19del) or leucine-to-arginine substitution in exon 21 (Leu858Arg) [3]. Small molecule EGFR tyrosine kinase inhibitors (EGFR-TKIs) are the first-line treatment for patients with EGFR mutation positive advanced NSCLC [4]. Interstitial lung disease, an adverse event of chemotherapy and EGFR-TKIs, may be fatal for patients with NSCLC who have interstitial pneumonia (IP), and it acts as a barrier to treatment in these patients [5,6]. According to previous trials that included patients with IP, combination therapy with EGFR-TKI and vascular endothelial growth factor (VEGF) inhibitors does not increase the incidence of interstitial lung disease in patients with EGFR mutation positive NSCLC [7,8]. We report a case of a patient with ex19del mutation positive NSCLC and IP associated with anti-aminoacyl transfer RNA synthetase syndrome who received treatment with erlotinib and ramucirumab.

## Case presentation

A 79-year-old man was admitted to our hospital with dyspnea on exertion lasting several months. He had no medical or family history, allergies, or medications; however, he was an ex-smoker who had smoked one pack per day for 30 years until the age of 50. His oxygen saturation (SpO2) was 92% under 2 L/min of nasal oxygen administration. His temperature, pulse rate, and blood pressure were 36.7°C, 96 beats/min, and 138/92 mmHg, respectively. The presence of mechanic’s hand sign, V neck sign, and Gottron’s papule were observed during physical examination; however, heliotrope rash or neuropathy was not observed. Fine crackles in bilateral lower lung zones were noted during lung auscultation. The white blood cell (WBC) count was 6.92 x 10^9^/L (reference range: 3.3-8.6 x 10^9^/L), and the lactate dehydrogenase (LDH), creatine phosphokinase (CPK), and aldolase levels were 217 IU/L (reference range: 124-222 IU/L), 51 IU/L (reference range: 59-248 IU/L), and 3.1 U/L (reference range: 2.7-5.9 U/L), respectively. The C-reactive protein (CRP), rheumatoid factor, and sialylated carbohydrate antigen KL-6 levels were elevated to 23.5 mg/L (reference range: 0-3.0 mg/L), 376 mg/L (reference range: 0-150 mg/L), and 715 x 10^3^ U/L (reference range: 0-500 x 10^3 ^U/L), respectively.

The patient tested positive for anti-aminoacyl transfer RNA synthetase (ARS) antibodies, and for the purpose of malignancy screening, the tumor marker test revealed cytokeratin 19 fragment (CYFRA), carcinoembryonic antigen (CEA), carbohydrate antigen 19-9 (CA19-9), and prostate-specific antigen levels of 5.5 x 10^3^ ng/L (reference range: 0-2.2 x 10^3^ ng/L), 2.0 x 10^3^ ng/L (reference range: 0-5.0 x 10^3^ ng/L), 5.7 x 10^3^ U/L (0-37 x 10^3^ U/L), and 1.23 x 10^3^ ng/L (reference range: 2.7 x 10^3^ ng/L), respectively.

**Figure 1:**
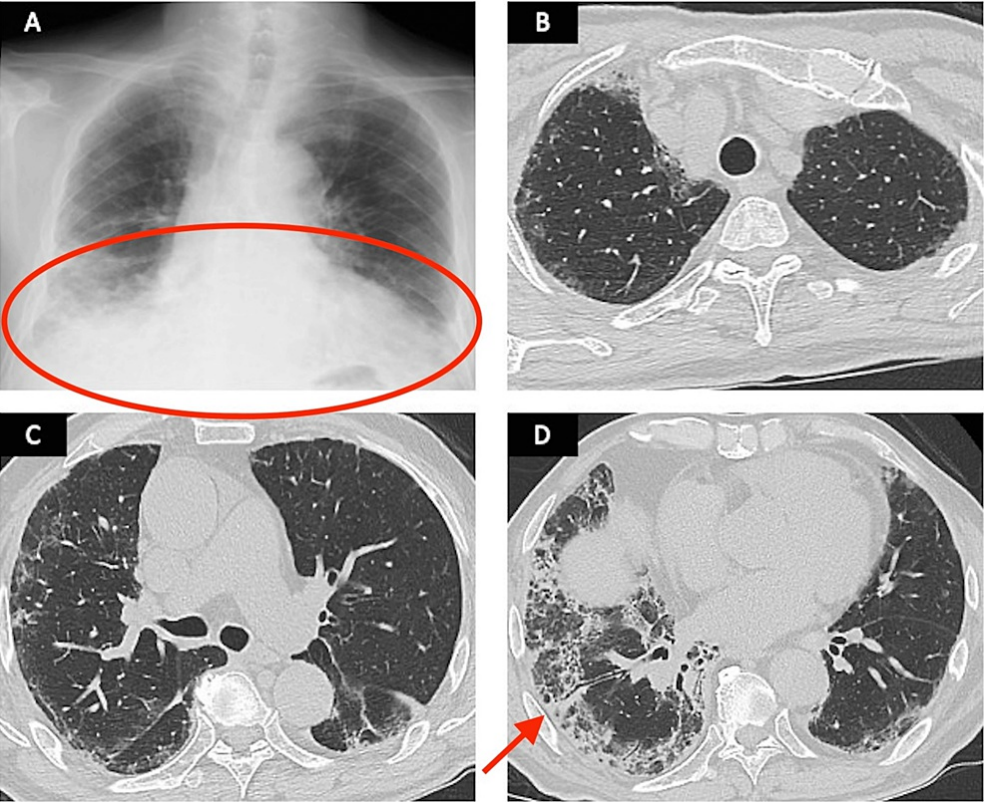
Initial diagnosis imaging examination (A) Chest radiograph showing patchy asymmetrical opacities in bilateral lower zones with septal thickening. (B–D) Chest CT showing bilateral ground-glass opacifications and mild traction bronchiectasis. CT, computed tomography

Chest radiography revealed the presence of patchy asymmetrical opacities in bilateral lower zones with septal thickening; however, no cardiomegaly or pleural effusions were observed (Figure 1a). Chest computed tomography (CT) revealed the presence of bilateral ground-glass opacifications and mild traction bronchiectasis, which were more pronounced in the lower lobes (Figures 1b-1d).

**Figure 2:**
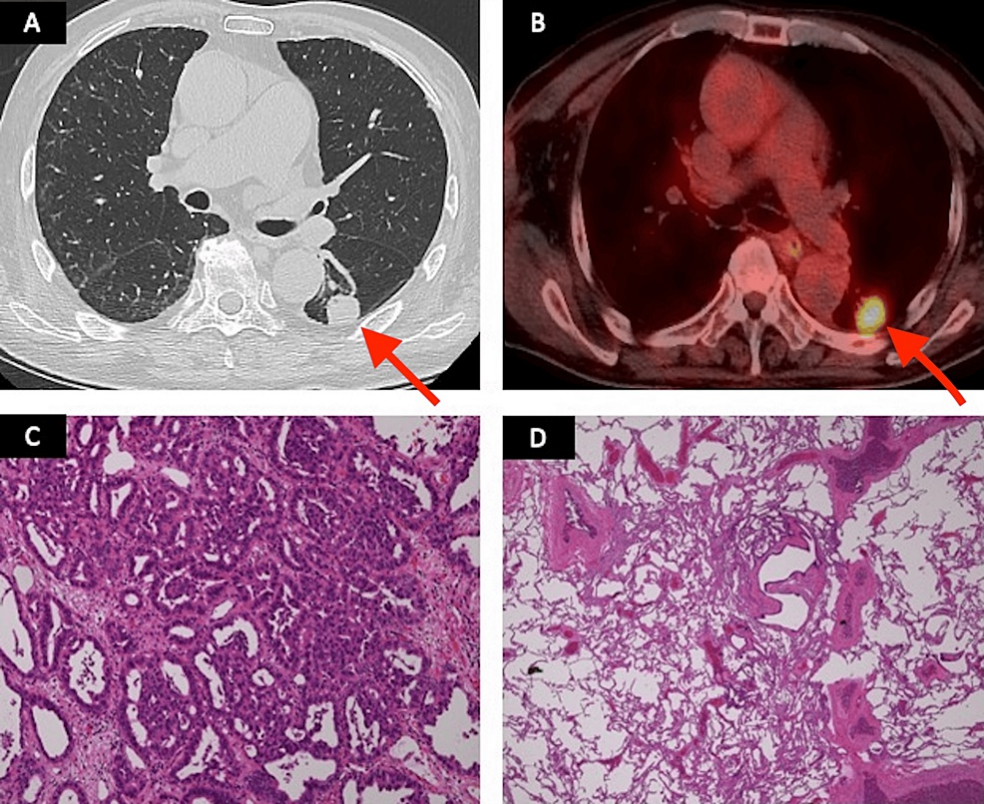
Preoperative imaging examinations and surgical specimen (A) Chest CT showing a 37 x 25 mm solid mass in the left lower lobe of the lung. (B) The maximum standardized uptake value was 6.4 during FDG-PET/CT, which is consistent with primary lung cancer with no evidence of metastatic disease. (C) Invasive adenocarcinoma with papillary and acinar patterns (hematoxylin and eosin staining x40). (D) Perivascular fibrosis and mild traction bronchiectasis (hematoxylin and eosin staining x40). CT, computed tomography; PET, positron emission tomography; FDG, fluorodeoxyglucose; SUVmax, maximum standardized uptake value

Based on these findings, the patient was diagnosed with anti-ARS antibody syndrome and treated with prednisolone and tacrolimus. The tumor marker CYFRA was elevated, and a solid mass measuring 37 x 25 mm was detected in the left lower lobe of the lung via CT during the outpatient patient visit (Figure 2a). The maximum standardized uptake value (SUVmax) was 6.4 on positron emission tomography (PET)/CT, which was consistent with primary lung cancer with no evidence of metastatic disease and lymphadenopathy noted (Figure 2b). Magnetic resonance imaging (MRI) of the head revealed no evidence of brain metastasis. CT-guided needle biopsy of the left lower lobe led to the detection of adenocarcinoma, and immunohistochemistry revealed that the cells were positive for cytokeratin (CK) 7, thyroid transcription factor 1 (TTF-1), and napsin A. A diagnosis of cT2aN0M0, stage ⅠB adenocarcinoma was made according to the American Joint Committee on Cancer, Eighth Edition. The patient was a candidate for surgical resection of the lung cancer.

Mild restrictive ventilation impairment was detected during the pulmonary function test, and the forced vital capacity (FVC) and total lung capacity (TLC) showed a slight decrease to 2.26 L and 3.74 L, respectively. Diffusion capacity for carbon monoxide (DLCO) and forced expiratory volume in 1 second (FEV-1) were within the normal range at 93.9% and 1.85 L, respectively. The dosage of prednisolone was reduced to 5 mg subsequently, and IP was considered stable. The patient underwent video-assisted thoracic surgery (VATS) of the left lower lobe with mediastinal lymph node dissection (intralobar, hilar, paratracheal, paraesophageal, and pulmonary ligament lymph nodes) for resection of the tumor, and the pathological stage of the tumor was classified as T2aN1M0, stage ⅡB adenocarcinoma. The surgical specimen was positive for only Ex19del (Oncomine Dx Target Test Multi-CDx system) but negative for PD-L1 (22C3). Pathological examination revealed the presence of perivascular fibrosis and mild traction bronchiectasis; however, IP was stable (Figures 2c, 2d). The patient had an ECOG performance status of 0, indicating eligibility for postoperative adjuvant chemotherapy based on the pathological stage; however, due to advanced age, the patient declined postoperative adjuvant chemotherapy and opted for observation.

**Figure 3:**
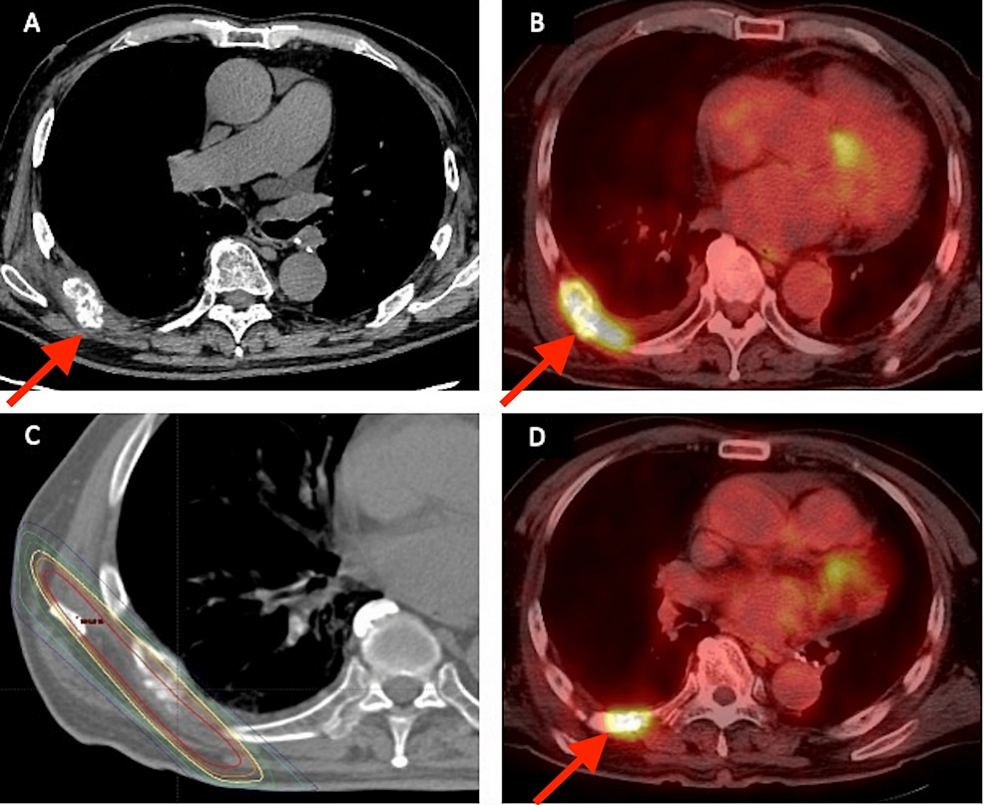
Imaging findings of right seventh rib metastasis and radiation therapy area (A) Chest CT showing a lytic lesion in the right seventh rib. (B) FDG-PET/CT showing abnormal FDG accumulation in the right seventh rib. (C) Radiation therapy (30 Gy in six fractions) for the right seventh rib metastasis. (D) FDG-PET/CT showing the recurrence at the edge of the irradiated field. CT, computed tomography; PET, positron emission tomography; FDG, fluorodeoxyglucose

The patient developed right chest pain three months after the surgery, and the chest CT revealed the presence of a lytic lesion in the right seventh rib. Abnormal fluorodeoxyglucose (FDG) accumulation in the same area was observed on FDG-PET/CT, indicating bone metastasis in the right seventh rib. Oligometastasis was suspected, and radiation therapy at a total dose of 30 Gy in six fractions was initiated (Figures 3a-3c). However, recurrence was observed at the edge of the irradiated field five months after receiving radiation therapy (Figure 3d). As re-irradiation was difficult, complicated by IP, a combination therapy of erlotinib and ramucirumab rather than osimertinib monotherapy was opted for.

**Figure 4:**
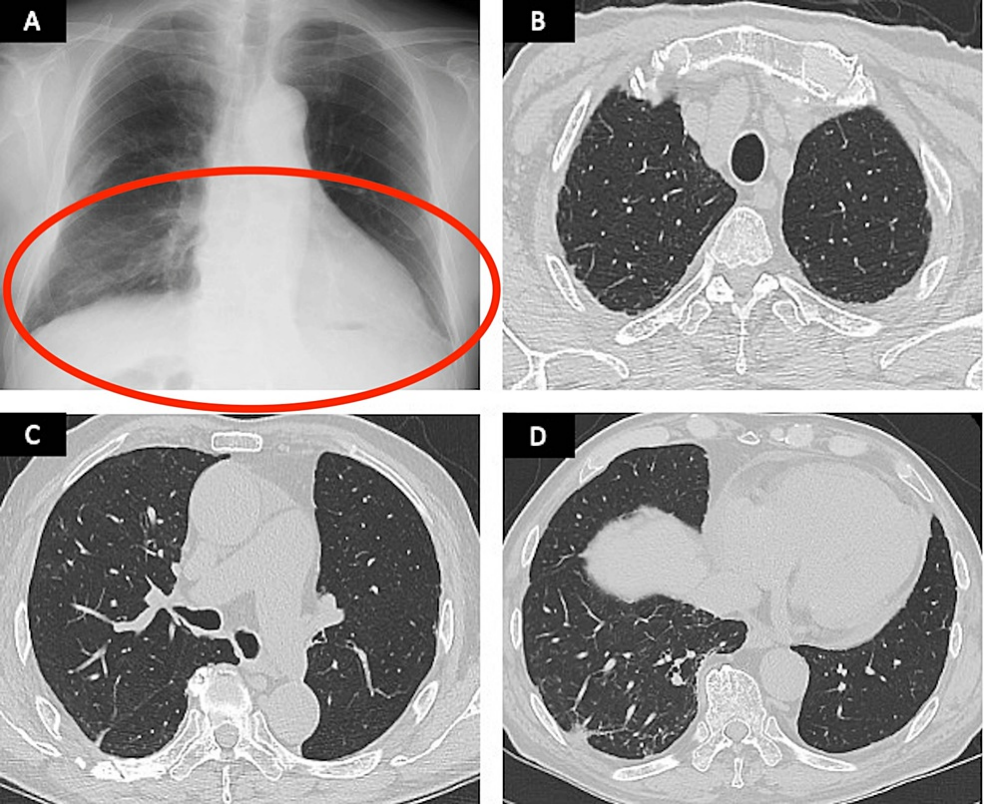
Imaging examination one year after the start of treatment (A) Chest radiograph showing mild reticular shadowing in bilateral lower zones. (B–D) Chest CT showing no recurrence of lung cancer and improvement of interstitial pneumonia. CT, computed tomography

Approximately one year has passed since the initiation of treatment, and acute exacerbation (AE) of IP has not been observed during the follow-up period. The tumor has remained stable, indicating stable disease (Figures 4a-4d).

## Discussion

We reported a case of a patient with ex19del-positive NSCLC and IP who received treatment with erlotinib and ramucirumab and achieved positive treatment outcomes without experiencing any AE of IP. The use of EGFR-TKIs is often avoided in patients with NSCLC who have IP. Patients with IP are more susceptible to developing lung cancer; however, the incidence of EGFR positivity is approximately 3%, which is lower than that of patients without IP [9,10]. The EGFR positivity rates differ between patients with honeycomb lung and those without it. The positivity rate in patients without honeycomb lung has been reported to be around 18%, which is similar to that of patients without IP [11]. Therefore, it is necessary to perform genetic testing, such as EGFR testing, and consider treatment options even in patients with IP.

Drug-induced pulmonary disease is an adverse event that may occur during lung cancer treatment. The reported incidence rates of pulmonary disease induced by EGFR-TKI (gefitinib) and conventional chemotherapy are 4.5% and 1.7%, respectively. The mortality rate of pulmonary disease induced by these drugs is high, at 31.6% and 27.9%, respectively; therefore, caution should be exercised during lung cancer treatment [12]. The risk factors for drug-induced lung disease include age over 60 years, pre-existing IP, history of lung surgery, reduced respiratory function, home oxygen therapy, history of radiation therapy to the lungs, use of multiple chemotherapy agents, and kidney dysfunction. In the present case, age, pre-existing IP, and history of lung surgery predisposed the patient to drug-induced lung disease. Pre-existing interstitial lung disease is a strong risk factor for the incidence of drug-induced pulmonary disease during lung cancer treatment [12]. AE occurs in 8.5% of patients with IP who do not have lung cancer, per year [13]. AE of IP due to treatment must also be considered a potential adverse event in patients with lung cancer who have IP. Among the patients receiving conventional chemotherapy (weekly paclitaxel in combination with carboplatin), 5.6% experienced AE [14]. Risk factors for AE due to chemotherapy include age <70 years, poor performance status, and usual interstitial pneumonia pattern [15]. However, none of these risk factors was applicable in the present case. It is difficult to distinguish between drug-induced pulmonary lung disease and AE of IP when a diffuse pulmonary injury occurs after chemotherapy in patients with lung cancer who have IP. In patients with diffuse pulmonary injury, administration of the drug must be discontinued, and steroid treatment must be initiated. In addition, a sufficient risk assessment must be conducted before treatment. Furthermore, it must be noted that the use of EGFR-TKI is not recommended in these patients.

AE of idiopathic pulmonary fibrosis (IPF) and non-specific pneumonia is associated with low survival rates. Although the pathology of AE features diffuse alveolar damage, which is also observed in AE of typical IP, its etiology has not been revealed yet. A heterogeneous increase in the density of alveolar capillaries in the lungs and a reduction in the dense collagen and myofibroblasts in the fibrotic lesion are observed in IPF. In addition, regenerated alveolar type Ⅱ epithelial cells produce VEGF and interleukin-8 in the angiogenesis zone, which leads to the proliferation and dilation of the alveolar capillaries. VEGF increases vascular permeability by impairing the endothelial cell junctions. A heterogeneous increase in the density of alveolar capillaries and vascular permeability may result in the occurrence of leaky lesions in AE [16,17], and it is possible that VEGF may have a similar effect in IPs other than IPF.

Bevacizumab is a humanized anti-VEGF monoclonal antibody that inhibits the vascular permeability of tumors, thereby improving drug delivery and increasing the drug concentration in the tumor. It is used in lung cancer treatment in combination with platinum-based chemotherapy or immune checkpoint inhibitors. A previous trial reported a significantly lower incidence of chemotherapy-related AE in the group that received bevacizumab in combination with first-line chemotherapy for lung cancer compared with that of the group that did not receive the combination [18]. In another trial, the incidence of treatment-related AE was reduced when bevacizumab was used in combination with osimertinib, a third-generation EGFR-TKI, in patients with ex19del or Leu858Arg-positive NSCLC, including those with a history of IP [7].

Ramucirumab is a human monoclonal IgG1 antibody that selectively targets VEGF receptor 2 and blocks signaling mediated by VEGF-A, VEGF-C, and VEGF-D. Thus, ramucirumab has a broader range of anti-tumor activity than other VEGF inhibitors. Ramucirumab, in combination with docetaxel, is indicated for the treatment of patients with metastatic NSCLC whose disease has progressed during or after receiving platinum-based chemotherapy. A trial on the use of combination therapy with EGFR-TKI and an inhibitor of VEGF reported that the combination of ramucirumab and erlotinib, a first-generation EGFR-TKI, extended progression-free survival and decreased the incidence of treatment-related AE in patients with a history of IP and those with ex19del or L858R-positive lung cancer [8].

Nintedanib is an intracellular inhibitor that targets multiple tyrosine kinases, including VEGF, fibroblast growth factor, and platelet-derived growth factor receptors. In the INPULSIS-2 trial, the time to the first AE was significantly prolonged in the nintedanib group compared with that in the placebo group. In addition, the proportion of patients who experienced AE was also significantly reduced in the nintedanib group [19]. These results suggest that VEGF may be involved in the AE of IP. Although treatment with nintedanib may be effective in this scenario, it has not been used in clinical practice as there is no progressive decline in pulmonary function over time, and AE of IP is not classified as progressive pulmonary fibrosis. Thus, although the use of VEGF antibodies is considered relatively safe in patients with NSCLC and a history of ILD, it should be avoided to limit the treatment options. It has been reported that the combination of nintedanib and chemotherapy prolongs overall survival in patients with NSCLC who have IPF [20]. However, further research must be conducted on the use of nintedanib in combination with chemotherapy or EGFR-TKIs in patients with NSCLC who have advanced pulmonary fibrosis.

This case report has a limitation. The observation period was short as it has only been about one year since initiating combination treatment with erlotinib and ramucirumab. AE of EGFR-TKI often occurs within four weeks of administration; therefore, careful observation is necessary.

## Conclusions

As the treatment for advanced NSCLC has rapidly evolved over the past few decades, numerous drugs are now used as standard therapy. Furthermore, EGFR-TKIs and immune checkpoint inhibitors have also been used in perioperative therapy in recent years. Thus, an increase in its use for the treatment of NSCLC with interstitial lung disease is predicted in the future. Going forward, further research must be conducted on these patients. This report will aid physicians in selecting treatments for patients with EGFR mutation positive advanced NSCLC.
